# Family planning interventions in Jordan: A scoping review

**DOI:** 10.1177/17455057231170977

**Published:** 2023-04-29

**Authors:** Rana AlHamawi, Yousef Khader, Mohannad Al Nsour, Raeda AlQutob, Eman Badran

**Affiliations:** 1 Global Health Development, Eastern Mediterranean Public Health Network, Amman, Jordan; 2 Jordan University of Science and Technology, Irbid, Jordan; 3 Department of Family and Community Medicine, School of Medicine, University of Jordan, Amman, Jordan; 4 Department of Pediatrics, Faculty of Medicine, University of Jordan, Amman, Jordan

**Keywords:** family planning, intervention, scoping review

## Abstract

**Background:**

Despite all efforts in Jordan to increase the demand and use of family planning services, many challenges have likely influenced fertility and contraceptive use outcomes. Improving accessibility and availability of family planning services and interventions to married women and their spouse is essential to improve pregnancy outcomes.

**Objectives:**

This study reviewed the gray and peer-reviewed literature published between January 2010 and June 2022 that described family planning interventions implemented in Jordan and highlighted the gaps identified in the literature.

**Eligibility criteria:**

For inclusion, primary studies that included information regarding family planning interventions implemented in Jordan were retained.

**Sources of evidence:**

PubMed database was searched between 2010 till June 2022, as well as bibliographies of the retrieved literature were screened for the relevant literature.

**Charting methods:**

Information extracted from the interventions included author, publication year, study design and purpose, intervention name, aim of the intervention, population descriptor and sample size of the intervention, and impact of the intervention.

**Results:**

A total of 10 studies that met the inclusion criteria were reviewed. The studies described/assessed 10 different interventions including communication interventions, child preparation programs, evidence-based educational program, counseling interventions, pharmacist booklet on effective use of oral contraceptive pills and Village Health Center project. Five family planning interventions targeted women and five targeted health care providers. Three interventions targeted men, two targeted religious leaders, and two targeted community health committees. Many of the interventions suffered from a lack of a robust methodological framework.

**Conclusion:**

This scoping review showed that there is scarce information on the implementation of High Impact Practices in Family Planning in Jordan. The review identified a lack of robust evidence on the impact and effectiveness of family planning interventions on the access to and use of family planning services and methods. There is a need for developing, implementing, and evaluating family planning interventions that elicit a positive environment and encourage the use of family planning services.

## Introduction

Family planning has many health benefits to women and the child as it contributes to birth spacing, reduces rates of elective abortions, as well as decreases the maternal and neonatal mortality rate associated with unintended pregnancy.^1,2^ Between the years 2012 and 2017–2018, the fertility rate in Jordan decreased to an average of 2.7 children per women, although still above the national replacement level of 2.1. The contraceptive prevalence rate in Jordan among married women aged 15–49 years increased from 40% in 1990 to 56% in 2002 and 61% in 2012. However, the increase has been almost entirely in the use of traditional methods.^3^ In 2019, half (52%) of married women used a method of family planning where 37% of married women aged 15–49 years used a modern method of family planning and 14% used a traditional method.^4^ Intrauterine devices (IUDs) were the most popular modern method, used by 21% of married women, followed by the pill (8%) and male condoms (5%). Among traditional methods, withdrawal was the most commonly used method where 13% of married women reported using it.^5^

Studies have shown that family planning services use and contraceptives uptake are determined by an interplay of factors such as socio-cultural and gender norms, misconceptions, poor knowledge of available services, and institutional and Health System Factors.^1,2^ Social and cultural norms, including preferences for large families, negatively affect the demand for family planning services among married couples.^6^

Improving accessibility and availability of family planning services and interventions to married women, as well as to men, is essential to decrease the fertility rate, delay first birth pregnancies, as well as improve pregnancy outcomes, maternal and child health and overall family’s health and social wellbeing.^7^ Delaying first pregnancy is of paramount importance because 15% of Jordanian women aged 25–49 years are married by the age of 18. In addition, 5% of women begin childbearing by age 15–19 years. Teenage childbearing is more common among women residing in Mafraq (13%), and among Syrian women residing in Jordan (28%).^5^ Adolescent mothers (aged 10–19) face higher risks of eclampsia, systematic infections, and puerperal endometritis than women aged 20–24 years, as well as children of adolescent mothers face higher risks of preterm birth, low birth weight, and severe neonatal condition.^8^ The risk of maternal and neonatal mortality can be decreased by as much as 40% by improving family planning interventions.^9^ A systematic review of the economic evaluation of family planning interventions in low- and middle-income countries reported that a decrease in the unmet need of family planning would be highly cost-effective.^7^

One study has collated the literature evidence regarding policies and programs related to family planning in Jordan;^10^ however, it focused on the literature targeting the youth population aged 10–24 years. It is critical to explore the available interventions that also target older populations and men. In addition, there is scarce information on the implementation of High Impact Family Planning Practices (HIPs) in Jordan. The HIPs are a set of evidence-based practices that reflect the global expert consensus of “what works in family planning” across the service delivery landscape, enabling environment and social and behavior change considerations.^11^ Therefore, this study aimed to review the gray and peer-reviewed literature published in the period January 2010—June 2022, to describe family planning interventions that have been implemented in Jordan and highlighted the gaps identified in the literature.

## Method

### Study design

A scoping review was conducted to describe family planning interventions in Jordan and identify the gaps in the research that studied these interventions. The PRISMA-ScR guidelines were followed when preparing the article. According to the Canadian Institutes of Health Research, scoping reviews are “exploratory projects that systematically map the literature available on a topic, identifying key concepts, theories, sources of evidence and gaps in the research.”^12^ Scoping reviews can be conducted for several reasons, with the most common being to map and summarize evidence, inform future research, and identify knowledge gaps. A preliminary search was conducted using PubMed database to be familiar with the literature, refine the aims and research question, and identify the relevant key words and Medical Subject Headings (MESH terms) to be used in the formal literature search. The search strategy was centered on the concept of family planning interventions. PubMed database was used to identify peer-reviewed articles that meet the eligibility criteria. Bibliographies of the retrieved literature were hand-searched to identify the relevant publications and/or gray reports that were cited. The search terms used are shown in Box 1.

**Box 1.** Search terms.

**Family Planning** 1. “Family Planning”[tw] OR “birth control”[tw] OR contraception[MeSH] OR “contraception postcoital”[MeSH] OR “contraception, barrier”[MeSH] OR “contraception, immunologic”[MeSH] OR “hormonal contraception”[MeSH] OR contracepti*[tw] OR “birth control”[tw] OR “natural family planning methods”[MeSH] OR 2. “Birth spacing”[tw] OR “birth intervals”[MeSH] OR 3. “Sex education”[MeSH] AND **Interventions** 1. *[tw] OR program*[tw] OR 2. “Family planning services”[MeSH] OR 3. “Family planning policy”[MeSH] OR policy[tw] AND **Jordan** 1. [MeSH]

### Inclusion criteria

Peer-reviewed papers that were published from January 2010 till June 2022 in journals indexed in PubMed were included in this study, as well as gray reports identified through citation searching. For inclusion, studies needed to include information regarding family planning interventions in Jordan, including interventions or programs or services focused on provision of family planning services, health promotion and education targeting communities or youth or married, pregnant women and/or single women in particular women in their child-bearing age, as well as married and/or single men. Interventions that involved communities or certain geographical locations, conducted in health care settings or outreach settings, as well as targeted a certain age group, were also included. Peer-reviewed primary research studies that encompass quantitative, qualitative, or mixed data were included. Opinion papers and letters, as well as secondary research were excluded as they are not relevant to the objective of our study. We have limited the time frame of our search for contextual relevance, as before the year 2010, there was limited services and knowledge regarding family planning in Jordan. The identified studies were imported into the Rayyan tool to facilitate the screening of studies. The Rayyan tool is a web and mobile application to facilitate the screening of articles for systematic and scoping reviews.^13^ Titles and abstracts were screened by the authors using the inclusion criteria for eligibility.

### Data extraction

An extraction form was developed and reviewed by the authors to ensure that all relevant information in relation to the research objectives was captured. The extraction form included as follows: author, publication year, study design and purpose, intervention name, aim of the intervention, population descriptor and sample size of the intervention, and impact of the intervention. Data were described in tabular format.

## Results

### Study characteristics

**Figure 1.**
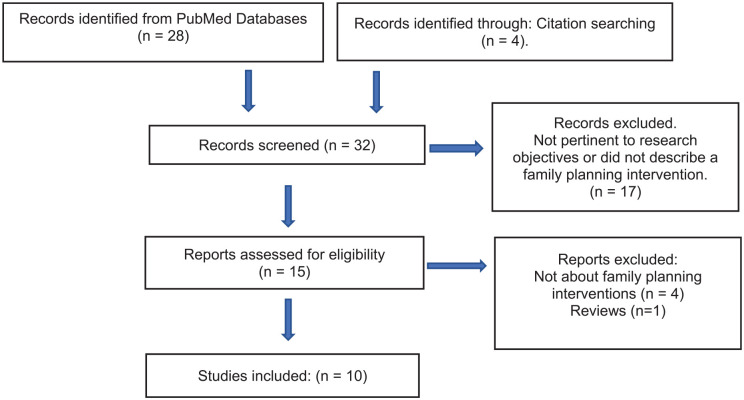
Flow chart for scoping review.

A total of 32 studies were identified and screened for eligibility. Of these, 22 studies were excluded because they were not pertinent to the research objectives or did not describe a family planning intervention. A total of 10 studies were included in this scoping review: 8 were peer-reviewed published articles and 2 were gray reports extracted from citation searching of eligible studies. The 10 studies described 10 interventions: 2 communication interventions targeting religious leaders, 3 childbirth preparation programs, 1 educational program tailored to reduce the provider’s misconception toward the injectable contraceptive Depot Medroxyprogesterone Acetate, 2 counseling interventions, 1 pharmacist booklet on the effective use of oral contraceptive pills, and 1 village health center (VHC) project. The flow chart in Figure 1 shows the number of studies included and excluded at each stage. The studies were published between 2012 and 2019. Three studies were quasi-experimental studies, three were experimental studies, and four were descriptive studies. Five family planning interventions targeted women,^13–17^ five targeted health care providers,^14,17–20^ three targeted men,^15–17^ two targeted religious leaders,^21,22^ and two targeted community health committees.^18,20^ The studies and interventions are summarized in Table 1.

**Table 1.** Summary of studies tailored at family planning interventions implemented in Jordan that were retrieved through PubMed from 2010 to June 2022.

Author, publication year	Study design/purpose	Family planning interventions	Aim of the intervention	Location of the intervention	Population, sample	Impact of family planning intervention
Khresheh et al.^14^	Exploratory, descriptive design and action research approach to investigate the feasibility and outcomes of introducing childbirth preparation programs in a sample of Maternal and Child Health centers in Jordan	Childbirth preparation program	To improve the knowledge of Jordanian women regarding childbearing practices and complications and ease anxiety and stress levels associated with childbearing experiences.	3 maternal and child health centers from 3 main regions in Jordan	107 primigravid women, six health staff (3 doctors and 3 midwives	• The number of women having normal births slightly increased compared with the national statistics • 88.9% exclusively breastfed for 2 months in comparison to 22.7% reported by national statistics • Increased knowledge of women and midwives about pregnancy, birth, and post-partum care • Promoted trust among women and health care providers • Provided women with a sense of control over childbirth and reinforced the importance of breastfeeding
Jordan Health Communication Partnership^15^	Cross-sectional study to study the impact of family planning material distributed to new parents on family planning use	Mabrouk I	To provide engaged couples who are about to get married with FP information	Civil Status and Passports Department (CSPD)	1016 individuals (513 men and 503 women)	• 35% of respondents in the Mabrouk I sample had heard about, seen, or read about the Mabrouk package • 84% of those who received Mabrouk I read it • 76% of those who read Mabrouk I reported discussing it with at least one other person • 96% of women who read the booklet, reported that they benefited from it. The self-reported unprompted benefits were that 71% learned about the importance of family planning, 50% about the importance of spousal communication and the importance of modern family planning methods • A slightly insignificant increase in Mabrouk I readers visiting MCH centers compared with non-readers • Readership status did not influence utility or intention to use modern contraceptive methods
Jordan Health Communication Partnership^16^	Cross-sectional study to study the impact of family planning material distributed to new parents on family planning use.	Mabrouk, you are the mother and father (Mabrouk II)	To provide new parents with information about family planning and birth spacing.	Civil Status and Passports Department (CSPD)	1217 individuals who received the booklet	• Approximately half of the respondents reported that they had ever heard of, seen, or read the package • 88% of those who viewed the booklet reported to have read the contents • About 92% benefited from the package: 21% learned about the importance of family planning after the first child, 14% about the importance of modern family planning methods, and 13% about the importance of postnatal care for the mother’s health • Three-quarters reported discussing the package messages with their spouse • Readership status did not influence the use or intention to use modern contraceptive or birth spacing
Akour et al.^13^	Randomized interventional study to examine the effect of a pharmacist-provided information booklet on improving the knowledge of Jordanian women about safe and effective oral contraceptive pills use	Pharmacist provided information booklet	To improve the knowledge of safe and effective use of OCP among women	Community pharmacies or fertility, obstetrics and gynecology outpatient clinics in Amman	160 adult women (⩾ 18 years) who have used oral contraceptive pills at least once. 80 women received the intervention and 80 women were allocated to the control group	• Mean knowledge score of women in the intervention group improved significantly from 1.76 at baseline to 5.00 (*p* < .000) at endline, immediately post intervention • The mean knowledge score of controls did not increase over time • Positive attitudes regarding family planning significantly improved from 5.15 to 5.50 in the intervention group, immediately post intervention • Attitudes of the control group did not significantly improve
El-Khoury et al.^17^	Randomized experimental study to evaluate the effects of involving men in family planning counseling	Family planning home-based counseling	To measure the effects of women-only counseling, couples counseling and no counseling on family planning use, knowledge, attitude and spousal communication about family planning	Al-Hashemi neighborhood in Amman, a low-income urban area	1247 randomly assigned to receive either women only counseling (417), couples counseling (416) or no counseling (414)	• Compared with no counseling, couples counseling led to an increase of 54% in the use of modern contraceptives. This increase is not statistically different from the increase attributed to women’s only counseling of 46% • Both women-only and couples counseling decreased the rate of traditional family planning methods by 7.3 and 8.3 percentage points, respectively, compared with controls • Women’s only and couples counseling improved family planning knowledge among women and men and spousal communication • Counseling reduced the number of women and men who expressed concern about the side effects of contraceptive methods • Counseling showed no significant impact on fertility preferences among men or women
Kamhawi et al.^18^	Cross-sectional study to describe the “consult and choose” program’s design and implementation and to assess the clients’ satisfaction with the program, as well as to assess from the clients perspective the providers’ adherence to the program	Consult and choose	• To equip health care providers with the necessary skills and tools to effectively interact with their clients • To assess whether health care providers adhered to the program	Maternal and Child Health Centers	• All Irbid family planning service providers • 461 women of reproductive age (15–49 years) who attended the clinic for family planning services at the period of recruitment	• Clients reported that providers performed 5.6 out of 7 of the steps in the protocol • Nearly 83% of respondents were very satisfied with their clinic visits • Between June 2011 and August 2012, 14,490 referral cards from community-based activities were collected in health centers, 59% of which were for family planning services • Service statistics trend indicate an increased number of new family planning users and in couple-years of protection after starting the CC program
El-Khoury et al.^19^	Randomized experimental study to assess the effects of an evidence-based medicine program on the knowledge, attitudes, and practices of healthcare providers	Evidence-based medicine program	To reduce health care provider’s misconception and bias toward the use of the contraceptive Depot Medroxy Progesterone Acetate (DMPA)	Amman and Zarqa	Population: 267 private doctors who provided family planning services. Sample: 267 private doctors, 135 in the treatment group and 132 in the control group	• No significant impact on provider’s knowledge of DMPA side effects or reported clinical practices • Increased positive attitude and confidence in prescribing DMPA, however insignificant
Komasawa et al.^20^	Quasi experimental difference in differences analysis to assess the impact of the VHC project on family planning use and effectiveness	Village health center (VHC) project	Aimed to enhance the quality and quantity of family planning services in VHCs	5 VHCs in Irbid	Household survey. Married women of reproductive age (15–49 years),1019 women in the intervention group and 1042 women in the control groups	• Compared with the control villages, women in the interventional villages had an increased use of VHC FP services and more participation in health promoting activities • 82.7% participants reported improvements regarding increased variety of services, 35.1% reported improvement in hard-ware setting, 34.6% reported improvement in IEC materials, and 51.4% reported a better attitude of nurse or midwives • An increase in counseling by VHC health care providers and a decrease in obtaining FP information from private clinics and TV
Underwood et al.^21^	Panel study to assess a communication program’s effectiveness in terms of knowledge regarding Islam’s approval of contraceptive methods, attitude change, and its impact on preaching and/or counseling of religious leaders	Communication training program	To enhance the role of RL in promoting family welfare, including reproductive health and family planning	Irbid	114 baseline and 94 endline men religious leaders; 22 baseline and 21 endline women religious leaders	• More religious leaders at endline cited more methods deemed acceptable according to Islamic teachings • Attitudes of RL regarding FP improved at endline • Religious leaders on average preached about more FP topics at endline, such as contraceptive methods, spousal communication, and Islamic religious opinion regarding FP
Underwood and Kamhawi^22^	Panel study design and baseline/endline quasi-experimental study to assess the impact of a communication intervention on religious leaders’ communication skills, and the secondary effect of the intervention on the mosque congregants	Communication training program	To enhance RL knowledge regarding family planning, their leadership skills, and to assess the secondary impact of the program on mosque congregants	Irbid (intervention group) Jerash (control group)	245 male and 145 female RL in Irbid. 420 intervention congregants and 420 control congregants	• RL post intervention reported a higher number of sermons or counseling on family planning, gender equity, reproductive health, and population issues compared with baseline data. • Congregants in the intervention group who recalled messages were more likely to act compared with control, such as, discussed messages positively with spouse/relatives/friends/neighbors/colleagues/family (65.7% vs 44.1%), encouraged their sons/daughters to use modern contraceptives (13.4% vs 4.2%), encouraged their sons/daughters to space between pregnancies (14.9% vs 2.5%), and decided to treat both male and female children equitably (40.3% vs 25.4%). • No significant change in attitude of the congregants of trained RLs compared with controls

### Childbirth preparation interventions

Three studies assessed childbirth preparation interventions.^14–16^ The first study was an exploratory descriptive study^14^ with an action research approach aimed to assess the impact of a childbirth preparation program (CBPP) on primigravid women, in particular, on their pregnancy and birth outcomes. The intervention was implemented in three randomly chosen Ministry of Health maternal and child health centers in the three main regions in Jordan. Healthcare providers were trained on the implementation of the intervention. The intervention provided a sample of primigravid women information regarding antenatal and postpartum care, physiological changes during pregnancy and postpartum, signs and symptoms of pregnancy complications, natural and medical pain management, stages of labor and methods of delivery, emotional changes (postpartum blues and postpartum depression), and family planning methods, advantages and disadvantages. Communication methods between healthcare providers and primigravid women included group discussions, role-play, brainstorming, visual aids, short videos, and demonstration and redemonstration techniques. A convenient sample of 107 primigravid women in their first trimester with uncomplicated pregnancies were chosen. Out of the 107 women initially enrolled in the program, only 36 fully completed the program. The study did not enroll a control group, instead outcomes were compared with the national Jordanian statistics, 2007.^23^ The study reported a slight increase in the rates of normal births (72.2%) in comparison to the Jordanian statistics (69.7%). However, the rates of exclusive breastfeeding in the first 2 months of the intervention was almost four times more compared with the Jordanian statistics. The findings from interviews concluded that the CBPP was effective in improving the knowledge of pregnancy, birth, and post-partum among women and midwives. Challenges to the implementation of the program were transportation difficulties, not getting their husbands’ permission to attend, long duration of the program, antenatal follow-up at a private clinic, as well as shortages of staff to implement the intervention and carry out their daily tasks.

The other two initiatives are implemented by the Civil Status and Passports Department (CSPD). The child preparation interventions are known as Mabrouk I^15^ and Mabrouk II.^16^ The Mabrouk I initiative targets couples who are engaged and are about to get married. Mabrouk II targets married couples who became parents for the first time. The packages include printed materials about the advantages of using modern contraceptives to space between pregnancies, the benefits of birth spacing, and the basic guidelines for childcare from birth through age 3.^15,16^ The Mabrouk II package includes additional information on postnatal care, child growth, gender equity, nutrition and vaccinations, and modern family planning methods. Couples should receive Mabrouk I and Mabrouk II packages when they visit the Civil Status and Passports Department (CSPD) to receive their family book or their first baby’s birth certificate, respectively. Mabrouk I and Mabrouk II packages were distributed starting on July 2008 and December 2008, by all 72 CSPD offices in the kingdom. To augment the distribution process, the CSPD incorporated a message reminding those generating their family book to pick up their copy of “Mabrouk” when they are notified that their family book is ready for pick-up via SMS text messaging.

The Mabrouk I^15^ and Mabrouk II^16^ initiatives were evaluated using telephone interviews and included a sample of recipients who registered their phone numbers at the CSPD upon receiving their family books or their first baby’s birth certificate. Mabrouk I was evaluated between December 2008 and December 2009, and Mabrouk II was evaluated between May 2011 and December 2011. Both studies reported that not everyone received the packages. However, the majority of individuals who received the packages read them and discussed the content with at least one other person. The majority of respondents said that they benefited from the package as follows: learned about the importance of family planning, spousal communication, or importance of modern family planning methods. Readers were recommended to distribute the package more widely and add more information about family health.

### Pharmacist booklet on effective use of oral contraceptive pills

A randomized interventional study^13^ examined the impact of an information-based booklet on the knowledge of effective and safe use of oral contraceptive pills among women. Adult women who used oral contraceptive pills as birth control at least once in their lifetime were recruited through a convenient sample of women who attended community pharmacies or fertility, obstetrics, and gynecology outpatient clinics in Amman. These women were then randomly assigned to either the intervention group (pharmacists-based booklet) or control group (traditional counseling). The booklet included information about the types of OCP, their mechanism of action, precautions and contraindications, advantages and disadvantages of each type of OCP, instructions about optimal use and potential drug–drug interactions, as well as information about alternative contraceptive methods. The booklet was developed by pharmacists, was six pages long, and took 10 min to explain to the participating women. The control group received conventional counseling about dosing regimens for OCP. All participants were interviewed three times by the pharmacist using structured questions to evaluate their knowledge and attitude change. The questionnaire was developed by the research team. For the intervention group, knowledge was evaluated before receiving the intervention, immediately after and 3 months post intervention. For the control group, knowledge was evaluated at the same time points but after receiving conventional counseling. The knowledge score was measured on a scale with a maximum score of 5. The score of the intervention group increased significantly from 1.76 at baseline to 5.00 immediately post intervention, but slightly decreased at follow-up to 4.93, while the control group showed no significant increase in knowledge over time. The attitude score was measured on a scale with a maximum score of 6. The attitude mean score of the intervention group improved significantly from baseline (5.15) to (5.50) immediately post intervention, while the control group showed no significant differences.

### Counseling

Two studies assessed the impact of family planning counseling on the uptake of modern family planning services^17^ and on client satisfaction with family planning visits.^18^ The first study^17^ assessed the impact of involving men in home-based counseling on family planning use, knowledge, attitudes, and spousal communication regarding family planning. The second intervention^18^ was implemented by the Jordan Health Communication Partnership (JHCP), which included counseling at health facilities and community-based activities to encourage women with unmet needs to visit health facilities that offer family planning services.

The first study^17^ described an intervention that was funded by USAID, known as Strengthening Health Outcomes through the Private Sector (SHOPS). The program provided home-based family planning counseling to married women, where trained female counselors made repeat home visits every 4–6 weeks for 6 months. Counselors were trained on couples counseling and effective communication strategies. During their visits, counselors discussed the benefits of family planning and birth spacing, addressed women’s concerns regarding specific methods, informed women of the modern methods available in Jordan, and made referrals to public and private service providers in the area. Women of low socioeconomic status, as assessed by the counselor, received project-funded vouchers for a free family planning service in selected private clinics. The voucher offered either three cycles of pills, 20 condoms, one 3-month injection, implant, or IUD insertion. In addition, the voucher covered the cost of one follow-up visit within a month of receiving the method. The voucher did not cover the cost of removing the implant or the IUD.

The study^17^ took place in the Al-Hashemi neighborhood of Amman, a low-income urban area that had not yet been covered by SHOPS. The researchers conducted a door-to-door household enumeration to identify eligible women: married, of reproductive age, living with their husbands, non-pregnant, fecund, not planning to move in the next year, and not using a modern family planning method. In all, 1247 women were randomly assigned to either women’s only counseling, couples counseling, or no counseling for a duration of 6 months. The study was implemented between September 2013 and August 2014. SHOPS-trained female counselors made repeat home visits every 4–6 weeks for 6 months. On average, women received three counseling visits (across both intervention arms), while men received one counseling visit (in the couples counseling intervention arm), as well as vouchers for family planning were provided to everyone enrolled in the study. Baseline and endline data were collected from women, and only endline data from men who participated. Counselors provided information regarding family planning benefits, availability of modern contraceptives, side effects of contraceptives, addressed concerns on specific methods, and made referrals to public and private service providers in the area. The study reported a 54% increase in the uptake of modern contraceptives due to couples counseling compared with the no counseling group. However, this was not significantly different from the 46% increase in the uptake of modern contraceptives in the women’s only counseling. The authors of the study did not separate the effect of the free vouchers from the effects of the counseling itself.

The second intervention^18^ is a client-centered family planning service known as consult and choose (CC) together with community-based activities. The aim of the intervention was to encourage women with unmet needs to visit health centers and enhance the quality-of-service provision by improving counseling skills of all health care providers in Irbid. The initiative followed a push–pull approach. The initiative involved community-level interventions that encouraged women to seek family planning services, as well as advocated for men’s support in their wives’ decisions (push factor). The initiative also equipped healthcare providers with the necessary skills and tools to effectively interact with their clients (pull factor). JHCP provided technical and financial assistance to nine community health committees to implement community-level interventions, such as, plays, debates, awareness sessions, and home visits, that discussed issues regarding contraceptive use, marriage and family health, and the importance of visiting health centers. In addition, all religious leaders (*n* = 776) in Irbid attended a 3-day training program that focused on family planning and highlighted Islam’s approval of family planning and modern contraceptives. Through sermons and religious lessons held in mosques, leaders were able to raise awareness regarding such topics and showcased their approval regarding modern contraceptives. In addition, JHCP provided all Maternal and Child health centers in Irbid with essential CC tools and skills to interact with clients effectively, such as follows:

- The Global Handbook for Family Planning Providers,^24^ as well as the World Health Organization (WHO’s)^25^ Medical Eligibility Criteria Wheel for Contraceptive Use;
- Service Provider Cue Cards for eight contraceptive methods that included a picture of the method, description of its effectiveness and how to use the method, major advantages and side effects, and how to manage side effects;
- Client Cue Cards for every modern method that the client takes home that included a picture and the name of the client’s chosen method, when to return, management of side effects, grace period if a dose is missed, pointers for a dialogue with one’s husband, and religious messages.

All Irbid family planning service providers in MCH were trained during a half a day session on the above-mentioned CC tools and reinforced proper counseling techniques.

The study^18^ conducted exit interviews with clients to assess the clients’ perspective on whether trained providers adhered to the CC protocol and used the CC tools; in addition, clients’ satisfaction regarding the initiative was measured. Clients on average reported that providers performed 5.6 of the 7 steps outlined in the CC protocol. Almost 20% of the respondents were dissatisfied with their clinic visits on the day of the interview. Client satisfaction with the clinic visits was strongly and positively associated with having a provider who, based on the client’s perception, followed the CC counseling protocol. The high rate of dissatisfaction might mean that not all health care providers utilized the CC tools effectively. Between June 2011 and August 2012, 14,490 referral cards from community-based activities were collected in health clinics.

### Evidence-based medicine program

The evidence-based medicine (EBM) program is part of the 5-year program (2005–2010), USAID-funded Private Sector Project for Women’s Health.^19^ The aim of the EBM program was to reduce provider’s misconception and bias toward the injectable contraceptive Depot Medroxyprogesterone Acetate (DMPA). The EBM program was evaluated using a single blinded experimental study design,^17^ where all gynecologists, obstetricians, and general practitioners who provided family planning services in the private sector in Amman and Zarqa governorates (totaling 267 doctors) were included. Healthcare providers were randomly assigned to the treatment group (135 doctors) and control group (132 doctors). The EBM program included a roundtable seminar and two one-on-one 15-min educational visits that reinforced the messages from the seminar, held in doctors clinics. The seminar was a 2-h session led by trained health care providers and included clinical research findings regarding DMPA. The control group was not invited to the seminar and was not offered education visits related to DMPA. However, it was offered two repeat Combined Oral Contraceptive (COC) pill educational visits from the previous year’s material, to keep them engaged during the study period. Survey findings showed no significant differences between the control group and the interventional group in knowledge change regarding DMPA side effects and clinical practices (prescribing or discussing DMPA with patients). However, it reported an improvement, although not significant in their attitudes toward DMPA, their confidence level in prescribing DMPA to patients and how knowledgeable they felt about DMPA. The authors have concluded that the EBM program is not effective as a stand-alone program for changing the patient’s and provider’s pervasive bias and misconception regarding DMPA. The study also suffered from selective participation, as treatment providers who attended the DMPA seminars were on average better informed and more positive toward DMPA than treatment providers who did not attend. Therefore, the authors concluded that the health care providers who would benefit most from the program did not participate in it.

### VHC project

A quasi-experimental study^20^ using differences in differences (DID) analysis was conducted. It assessed an intervention called “Village Health Center Project.” The VHC project equipped 14 VHCs located in rural areas, away from primary or comprehensive health centers (PHC/CHC), with the essential equipment to carry out family planning services. The study aimed to assess the impact of the project in five intervention VHCs in Irbid. The study matched each intervention VHC to a control VHC in the same health district, for demographic and economic relevance. The intervention comprised two components: Community-based interventions and Facility based interventions. The facility-based interventions included four training sessions for nurses or midwives working full-time at the VHC, three workshops for part-time doctors and midwives working at VHCs, provided VHC with basic medical equipment, furniture, information, education, and communication (IEC) materials, materials required for family planning services (such as contraceptive pills and male condoms), conducted supervisory visits by maternal and child health supervisors from a local health directorate or MoH, and updated the family planning service manual at the VHC. The community-based interventions included the following components: supported the establishment of a community health committee in each village, conducted workshops for each committee, encouraged committees to develop family planning action plan, monitored committees’ activities each month, and provided seed money for the first 4 months of the project. The study results reported several outcomes in the following categories: family planning service use in the centers, the women’s participation in health promotion activities, and their source of reproductive health information. In total, 510 and 509 women at baseline and 508 and 534 at endline were enrolled in the intervention and control groups, respectively. Regarding the use of family planning services at VHCs, the intervention effects were for the following indicators: obtaining contraceptives, family planning counseling, and general counseling increased significantly by +7.6 pp, +4.1 pp, and +4.9 pp with *p* < 0.001, *p* = 0.004, and *p* = 0.005, respectively. The second category included two indicators: participation in education sessions in VHCs and participation in community health activities. The education sessions at VHCs and community health activities had intervention effects of +11.5 pp and +8.1 pp, respectively, with *p* < 0.001 for both. Regarding the source of information, counseling by VHC staff showed an upward trend, while counseling by private doctors showed a downward trend for both the intervention and control groups.

### Communication intervention targeting religious leaders

There are two baseline/endline quasi-experimental studies^21,22^ investigating the effect of a communication training program in improving the knowledge of family planning and leadership skills of Muslim religious leaders in Irbid. Both studies^21,22^ aimed to assess the knowledge change of religious leaders following the intervention as well as the change in their preaching and counseling frequencies regarding family planning topics such as gender equity, modern contraceptives, birth spacing, and reproductive health. Underwood and Kamhawi^22^ also assessed the secondary impact of the intervention on mosque congregants. The communication interventions for both studies were based on the Religious Leaders Training Manual on Family Health, which provided in-depth and cultural appropriate information regarding family’s welfare, Islam and family health, gender equity, safe motherhood, family planning methods, birth spacing, and incorporated authentic Islamic sources of Prophet Mohammad’s saying and related actions to family planning. The communication program is composed of a 2-day workshop with eight sessions where religious leaders are gathered in groups of 20–25 individuals. During the workshop, participants developed their own action plan that will facilitate the dissemination of learnt information regarding family planning in either Friday sermons, religious lessons, or counseling sessions. Both studies showed significant improvements in religious leaders’ knowledge of family planning topics at endline compared with baseline data, as well as showed increased frequency of preaching about family planning. A study by Underwood and Kamhawi^22^ examined the secondary impact of the intervention on mosque congregants, using a control group. While the study reported less recollection of messages in the intervention group compared with the control group, the congregants in the intervention group who recalled the messages were more likely to take action related to the topics to which they were exposed to, such as discussed messages positively with spouse/relatives/friends/neighbors/colleagues/family (65.7% vs 44.1%), encouraged their sons/daughters to use modern contraceptives (13.4% vs 4.2%), encouraged their sons/daughters to space between pregnancies (14.9% vs 2.5%), and decided to treat both male and female children equitably (40.3% vs 25.4%).

## Discussion

Scoping reviews are intended to not just review what research has done, but where there are gaps in the research. Four studies assessed the HIPs across enabling environment including leading and managing rights-based family planning programs,^19^ policy processes,^20^ and social accountability to improve family planning information and services.^21,22^ Only one study assessed one service delivery HIP “Pharmacies and Drug Shops..”^13^ The rest of the studies assessed social and behavior change HIPs, including promoting healthy couples’ communication^17^ and knowledge, beliefs, attitudes, and self-efficacy.^14–16,18^ Evidence on the impact of other HIPs is lacking. Moreover, no evidence is available on enhancement HIPs including FP vouchers, adolescent-responsive contraceptive services, and digital health. Digital technologies such as mHealth for family planning have been shown to yield time and resource efficiencies for health care providers, as well as improve quality of care and hence result in better patient outcomes.^26,27^ A wide knowledge and research gap on postpartum family planning interventions were identified through this scoping review and little is known about interventional study designs. Therefore, this scoping review highlights the dearth of research on HIPs in Jordan.

It was evident in our scoping review that there is limited and lack of robust evidence regarding immediate postpartum care and postpartum interventions. Only one intervention known as the childbirth preparation aimed to provide postpartum care to primigravid women.^14^ Despite the positive impact of the program on women’s knowledge regarding postpartum care and improvement in exclusive breastfeeding in the first 6 months, the results were compared with national statistics due to the lack of a control group, even though the sample was not representative of the Jordanian population. The study initially enrolled 107 women, but only 36 fully completed the program.^14^ Therefore, the validity of these findings is limited by the high rate of attrition of participants (66%) and the findings should be interpreted with caution. Despite these limitations, increasing the duration of exclusive breastfeeding has a direct effect on increasing postpartum family planning use, as women who breastfeed exclusively benefit from the Lactational Amenorrhea Method which can play a role in lengthening birth intervals.

The second intervention that aimed to increase the knowledge of new parents about postpartum care is Mabrouk II.^16^ Despite the positive outcomes/results reported by Mabrouk II, not all participants who were contacted received the books, as well as not all participants who registered left their phone numbers at the Civil Status and Passport Department.^16^ These findings were also reported in the Mabrouk I document, which is phase I of the Mabrouk intervention.^15^ Lessons must be learnt from previous studies and wide dissemination of the books by the CSPD is critical for increasing the provision and knowledge of family planning services.

Embedded misconception and bias about family planning is apparent in Jordan.^28–30^ Enlisting a positive environment toward family planning is important for averting such constrictive beliefs.^10^ Two interventions were tailored at religious leaders to improve their knowledge and leadership skills to disseminate family planning information to congregants through Friday sermons, and religious and counseling sessions.^21,22^ Despite positive improvement in religious leaders’ knowledge regarding family planning, the attitudes of congregants of religious leaders did not improve compared with the control, as well as they were not more likely to recall family planning messages.^22^ No interventions were tailored at improving parents of married couples’ family planning knowledge and shifting their misconceptions and biases regarding reproductive health, even though pervasive restrictive beliefs about gender norms, contraceptive use, and fertility persist in Jordan.^28–30^ Despite this, young people perceive parents as a trustworthy source of reproductive health information.^10^ The EBM was seen as not suitable as a standalone method to target providers and clients’ misconception regarding DMPA.^19^ However, EBM was seen to improve knowledge and provider-related practices regarding Combined Oral Contraceptives (COC) and Progestogen-only pill (POP), such as stocking COC/POP, discussing COC/POP with patients, and prescribing COC/POP.^19^ Previous experimental evaluations^31–34^ of EBM programs related to other interventions, such as heart diseases, smoking cessation, cancer, and pain management have shown mixed results. Regarding DMPA findings, the fact that providers were resistant to adjust their own attitudes and clinical practices might be because of the low demand for DMPA, as well as providers and patients’ concerns regarding its side effects. DMPA is known to engender menstrual bleeding changes. Many women fear that menstrual changes, such as prolonged bleeding, heavier bleeding, spotting, irregular bleeding, and amenorrhea can lead to negative health consequences, including infertility.^35^ Evidence has shown that changes in menstrual bleeding associated with contraceptive use, can lead to discontinuation and nonuse of the contraceptive. The family planning counseling tool “NORMAL,”^36^ which was developed by FHI 360 and Population Services International (PSI), is a resource that health care providers could use to educate women on bleeding changes associated with contraception, address common misconceptions and fears about menstrual bleeding changes, and improve women’s awareness regarding the potential benefits of reduced menstrual bleeding and/or amenorrhea. This tool could be incorporated into family planning counseling sessions to avert misconceptions regarding menstrual bleeding changes associated with contraceptive use among women.

Our study is limited by the fact that only one database was searched, meaning that other eligible articles not indexed in PubMed were missed. In addition, our study search strategy might not have extracted all relevant studies from PubMed, therefore we might have missed some pertinent studies.

## Conclusion

This scoping review showed that there is scarce information on the implementation of HIPs in Jordan. The review identified limited and a lack of robust evidence on the impact and effectiveness of family planning interventions on the access to and use of family planning services and methods. There is a need for developing, implementing, and evaluating family planning interventions that elicits a positive environment and encourages the use of family planning services and methods, as well as averts bias and misconceptions regarding family planning among communities. Targeting parents with family planning information is needed to encourage young people to demand and utilize family planning services, as well as reinforce positive beliefs about family planning. Digital technologies such as mHealth for family planning must be assessed for effectiveness, as well as for feasibility in increasing family planning services/methods uptake.

## Supplemental Material

sj-docx-1-whe-10.1177_17455057231170977 – Supplemental material for Family planning interventions in Jordan: A scoping review Click here for additional data file. Supplemental material, sj-docx-1-whe-10.1177_17455057231170977 for Family planning interventions in Jordan: A scoping review by Rana AlHamawi, Yousef Khader, Mohannad Al Nsour, Raeda AlQutob and Eman Badran in Women's Health

## Declarations

**Ethics approval and consent to participate:** Not Applicable.

**Consent for publication:** Not Applicable.

**Availability of data and materials:** Not Applicable.

## References

[1] Tsui AO McDonald-Mosley R Burke AE. Family planning and the burden of unintended pregnancies. Epidemiol Rev 2010; 32(1): 152–174. 20570955 10.1093/epirev/mxq012 PMC3115338

[2] World Health Organization. Family planning/contraception methods: key facts, http://www.who.int/mediacentre/factsheets/fs351/en/ (accessed 27 June 2022).

[3] Bietsch K Arbaji A Mason J , et al. Shifting dynamics: changes in the relationship between total fertility rate and contraceptive prevalence rate in Jordan between 2012 and 2017. Gates Open Res 2020; 4: 160. 34345798 10.12688/gatesopenres.13188.1 PMC8282988

[4] Almalik M Mosleh S Almasarweh I. Are users of modern and traditional contraceptive methods in Jordan different? East Mediterr Health J 2018; 24(4): 377–384. 29972232 10.26719/2018.24.4.377

[5] Department of Statistics, Jordan and ICF. Population and family health survey 2017-2018, www.DHSprogram.com (accessed 27 June 2022).

[6] Ministry of Health. Family planning costed implementation plan (FP CIP): 2020-2024, https://pdf.usaid.gov/pdf_docs/PA00WB19.pdf (accessed 8 March 2023).

[7] Zakiyah N van Asselt AD Roijmans F , et al. Economic evaluation of family planning interventions in low and middle income countries; a systematic review. PLoS ONE 2016; 11(12): e0168447. 27992552 10.1371/journal.pone.0168447 PMC5167385

[8] World Health Organization. Adolescent pregnancy, https://www.who.int/news-room/fact-sheets/detail/adolescent-pregnancy (accessed 3 February 2023).

[9] Cleland J Conde-Agudelo A Peterson H , et al. Contraception and health. Lancet 2012; 380(9837): 149–156. 22784533 10.1016/S0140-6736(12)60609-6

[10] Gausman J Othman A Dababneh A , et al. Landscape analysis of family planning research, programmes and policies targeting young people in Jordan: stakeholder assessment and systematic review. East Mediterr Health J 2020; 26(9): 1115–1134. 33047803 10.26719/emhj.20.018

[11] The High Impact Practices Partnership (HIPs). Family planning high impact practices list. Washington, DC: The High Impact Practices Partnership, 2022.

[12] Canadian Institutes of Health Research. A guide to knowledge synthesis: a knowledge synthesis chapter, https://cihr-irsc.gc.ca/e/41382.html (accessed 5 January 2023).

[13] Akour A Bardaweel S Awwad O , et al. Impact of a pharmacist-provided information booklet on knowledge and attitudes towards oral contraception among Jordanian women: an interventional study. Eur J Contracept Reprod Health Care 2017; 22(6): 459–464. 29300110 10.1080/13625187.2017.1412425

[14] Khresheh R Almalik M Owies A , et al. Implementation of a childbirth preparation program in the maternal and child health centres in Jordan. Midwifery 2018; 61: 1–7. 29505945 10.1016/j.midw.2018.02.010

[15] Jordan Health Communication Partnership. Evaluation of the “Mabrouk” initiatives in the civil status and passports department (CSPD) offices, https://pdf.usaid.gov/pdf_docs/PBAAC322.pdf

[16] Jordan Health Communication Partnership. Evaluation of the “Mabrouk II: you’ve become a mother and a father” initiative, https://pdf.usaid.gov/pdf_docs/PBAAC324.pdf

[17] El-Khoury M Thornton R Chatterji M , et al. Counseling women and couples on family planning: a randomized study in Jordan. Stud Fam Plann 2016; 47(3): 222–238. 27611319 10.1111/sifp.69

[18] Kamhawi S Underwood C Murad H , et al. Client-centered counseling improves client satisfaction with family planning visits: evidence from Irbid, Jordan. Glob Health Sci Pract 2013; 1(2): 180–192. 25276531 10.9745/GHSP-D-12-00051 PMC4168569

[19] El-Khoury M Thornton R Chatterji M , et al. Effectiveness of evidence-based medicine on knowledge, attitudes, and practices of family planning providers: a randomized experiment in Jordan. BMC Health Serv Res 2015; 15: 1–9. 26431847 10.1186/s12913-015-1101-z PMC4592549

[20] Komasawa M Yuasa M Shirayama Y , et al. Impact of the village health center project on contraceptive behaviors in rural Jordan: a quasi-experimental difference-in-differences analysis. BMC Public Health 2019; 19(1): 1415. 10.1186/s12889-019-7637-9 PMC6820982 31664981

[21] Underwood C Kamhawi S Nofal A. Religious leaders gain ground in the Jordanian family-planning movement. Int J Gynaecol Obstet 2013; 123(Suppl. 1): e33–e37. 23987734 10.1016/j.ijgo.2013.07.006

[22] Underwood CR Kamhawi SS. Friday sermons, family planning and gender equity attitudes and actions: evidence from Jordan. J Public Health 2015; 37(4): 641–648. 10.1093/pubmed/fdu090 25395604

[23] Jordan Department of Statistics. Jordan population and family health survey 2007, https://dhsprogram.com/pubs/pdf/fr209/fr209.pdf

[24] World Health Organization. Family planning: a global handbook for providers, https://www.who.int/publications/i/item/9780999203705

[25] World Health Organization. Medical eligibility criteria wheel for contraceptive use, https://www.who.int/publications/i/item/9789241549257 (accessed 26 June 2022).

[26] Agarwal S Perry HB Long LA , et al. Evidence on feasibility and effective use of mHealth strategies by frontline health workers in developing countries: systematic review. Trop Med Int Health 2015; 20(8): 1003–1014. 25881735 10.1111/tmi.12525 PMC4692099

[27] World Health Organization. Recommendations on digital interventions for health system strengthening, https://www.who.int/publications/i/item/9789241550505 (accessed 28 January 2023). 31162915

[28] West L Isotta-Day H Ba-Break M , et al. Factors in use of family planning services by Syrian women in a refugee camp in Jordan. J Fam Plan Reprod Health Care 2017; 43(2): 96–102. 10.1136/jfprhc-2014-101026 26962045

[29] Shakhatreh FM. Family planning in women of childbearing age in disadvantaged south Jordan. Eur J Contracept Reprod Health Care 2012; 17: S72.

[30] Shattnawi KK Khader YS Al-Sheyab N , et al. Perceived barriers of using modern family planning methods among women in Jordan: a qualitative study. Int J Community Based Nurs Midwifery 2021; 9(4): 278–288. 34604397 10.30476/ijcbnm.2021.88675.1531 PMC8479286

[31] Dietrich AJ O’Connor GT Keller A , et al. Cancer: improving early detection and prevention. A community practice randomised trial. Brit Med J 1992; 304(6828): 687–691. 1571644 10.1136/bmj.304.6828.687 PMC1881551

[32] Katz DA Brown RB Muehlenbruch DR , et al. Implementing guidelines for smoking cessation: comparing the efforts of nurses and medical assistants. Am J Prev Med 2004; 27(5): 411–416. 15556742 10.1016/j.amepre.2004.07.015

[33] Feldman PH Murtaugh CM Pezzin LE , et al. Just-in-time evidence-based e-mail “reminders” in home health care: impact on patient outcomes. Health Serv Res 2005; 40(3): 865–886. 15960695 10.1111/j.1475-6773.2005.00389.x PMC1361172

[34] McDonald MV Pezzin LE Feldman PH , et al. Can just-in-time, evidence-based “reminders” improve pain management among home health care nurses and their patients? J Pain Symptom Manage 2005; 29(5): 474–488. 15904750 10.1016/j.jpainsymman.2004.08.018

[35] Polis CB Hussain R Berry A. There might be blood: a scoping review on women’s responses to contraceptive-induced menstrual bleeding changes. Reprod Health 2018; 15(1): 1–7. 29940996 10.1186/s12978-018-0561-0 PMC6020216

[36] Rademacher KH Sergison J Glish L , et al. Menstrual bleeding changes are NORMAL: proposed counseling tool to address common reasons for non-use and discontinuation of contraception. Glob Health Sci Pract 2018; 6(3): 603–610. 30287535 10.9745/GHSP-D-18-00093 PMC6172120

